# Advances in the ultrasound diagnosis in equine reproductive medicine: New approaches

**DOI:** 10.1111/rda.14192

**Published:** 2022-07-15

**Authors:** Cristina Ortega‐Ferrusola, Vanesa Gómez‐Arrones, Francisco E. Martín‐Cano, Mari Cruz Gil, Fernando J. Peña, Gemma Gaitskell‐Phillips, Eva Da Silva‐Álvarez

**Affiliations:** ^1^ Laboratory of Equine Reproduction and Equine Spermatology, Veterinary Teaching Hospital University of Extremadura Cáceres Spain; ^2^ CENSYRA, Centro de selección y reproducción animal de Extremadura Badajoz Spain

**Keywords:** 3D ultrasound, grey scale image, mare, power and colour Doppler, pregnancy diagnosis, pulse Doppler, stallion, subfertility

## Abstract

Ultrasound technology has led to new lines of research in equine reproduction, and it has helped to greatly improve clinical diagnosis and reproductive outcomes in equine practice. This review aims to discuss the potential clinical uses and new approaches of ultrasonography in equine reproduction. Doppler modalities are usually used to evaluate the vascularization of the follicles, corpus luteum (CL), and the uterus in the mare for diagnostic purposes. Inclusion of Doppler ultrasound in artificial insemination and embryo transfer programs could improve the reproductive outcome of these techniques. Better selection of recipients based on CL functionality, early pregnancy diagnosis 7–8 days postovulation of the donor before flushing or diagnosis of mares with endometritis with pathological increases of blood flow are examples of clinical applications in the mare. In the stallion, colour Doppler ultrasound has improved the diagnostic potential of B‐mode ultrasound, improving the differential diagnosis of pathologies such as testicular torsion (decrease or absence of blood flow in the cord) and orchitis (increased blood flow in the cord). The incorporation of pulsed Doppler ultrasound into the reproductive evaluation of the stallion has enabled early identification of stallions with testicular dysfunction, thus allowing administration of timely treatment and subsequent improvements of the fertility prognosis for these animals. In addition, this technique has been used in the monitoring of patients undergoing medical and surgical treatments, thus verifying their efficacy. Recently, computer‐assisted pixel analysis using specific software has been performed in research work in order to semi‐quantitatively evaluate the vascularization (colour and power Doppler) and echotexture of different organs. These softwares are now being developed for clinical purposes, as is the case with Ecotext, a computer program developed for the evaluation of testicular echotexture, providing information on testicular functionality.

## 1 INTRODUCTION

Ultrasound technology has significantly contributed to knowledge of many physiopathological aspects of equine reproduction, which has helped to greatly improve clinical diagnosis and reproductive outcomes in equine practice (Ginther, 2014; Ortega‐Ferrusola et al., 2014). Ultrasonography has become the most important tool for breeding soundness evaluation (BSE) in both mares and stallions and is an excellent tool for diagnosing various pathological disorders (Bollwein et al., 1998; Bollwein, Mayer, et al., 2002; Pozor, 2005; Pozor & McDonnell, 2004). However, its use is not very widespread among equine clinical practitioners in the field of reproduction, despite the fact that most portable equipment includes Doppler modalities. The relative lack of knowledge by practitioners about its potential applications to evaluate reproductive functions continues to limit its use in reproductive practice (Samir et al., 2021).

Different ultrasound modalities have been used to evaluate the reproductive tract in equines. B‐mode ultrasound provides information regarding anatomical features of different organs and their echotexture and allows the measurement of structures (Ginther & Utt, 2004). Doppler modalities have allowed study of testicular, uterine, and ovarian vascular hemodynamics in this species and this has contributed to knowledge of both physiological and pathological aspects of these organs (Bollwein, Mayer, et al., 2002; Bollwein, Weber, et al., 2002; Boyd et al., 2006; Pozor & McDonnell, 2004). These modalities have improved the diagnostic potential of conventional ultrasound, helping in the early identification of certain pathologies related to the perfusion of these organs and allowing monitoring of medical or surgical treatments (Bailey et al., 2012; Gracia‐Calvo et al., 2015; Savaş et al., 2002).

Doppler modalities work by providing a representation of blood flow detected. Colour Doppler uses a combination of a colour representation of blood flow with B‐mode imaging to allow qualitative assessment of a specific organ or area. Colour coding intensity allows subjective evaluation of flow velocities and flow direction (Ginther, 2007; Ginther & Utt, 2004). The extent of colour‐coding intensity can be estimated by percentage of a tissue with colour signals or can be calculated using external software, which assesses the number of coloured pixels (Delorme et al., 1995).

Power Doppler is also a qualitative technique, but with greater sensitivity than colour Doppler, allowing the detection of small diameter vessels with slower flow such as microvascularization of the endometrium, testicular parenchyma and corpus luteum (CL) (Ginther & Utt, 2004). This technique reflects the number of erythrocytes in a vascular segment. Therefore, the signal strength at each point is related to the number of blood cells that are moving per unit of time. Unlike colour Doppler, this modality is independent of the angle of insonation and does not provide information about the velocity or direction of blood flow. The computerized analysis of power Doppler images of organs such as the uterus, has allowed us, for example, to differentiate pregnant from non‐pregnant mares 8 days after ovulation and prior to embryo recovery (Nieto‐Olmedo et al., 2020).

Computer‐assisted pixel analysis using Image J software has been performed in research work in order to semi‐quantitatively evaluate the vascularization and echotexture of different organs based on the area and intensity of pixels identified in the image (B‐mode, colour or power Doppler) providing an indication of blood flow area in the image assessed or a grade of echogenicity (Delorme et al., 1995). Initially, this software has been used for research purposes, because the post‐acquisition image processing does not allow real‐time decisions to be taken. However, they are now being developed for clinical purposes, as is the case with Ecotext (Humeco, Huesca, Spain), a computer program developed for the evaluation of testicular echotexture in various species, providing information on testicular functionality (Abecia et al., 2020; da Silva‐Álvarez et al., 2022).

Finally, Spectral Doppler is the only modality that allows objective quantification of blood flow in a given vessel in real time and provides an objective set of flow velocities (peak systolic velocity (PSV), end‐diastolic velocity (EDV) and time average mean velocity (TAMV)) and peripheral vascular resistance parameters such as resistance index (RI: PSV ‐ EDV/PSV) and pulsatility index (PI: PSV ‐ EDV/MV) (Ginther & Utt, 2004). In general terms, Doppler indices increase in ischemic or degenerative processes and decrease in inflammatory conditions due to hyperaemia (Pozor & McDonnell, 2004). Each modality provides valuable information to the clinician, helping to contribute to diagnosis and management of various reproductive disorders and technologies.

**FIGURE 1:**
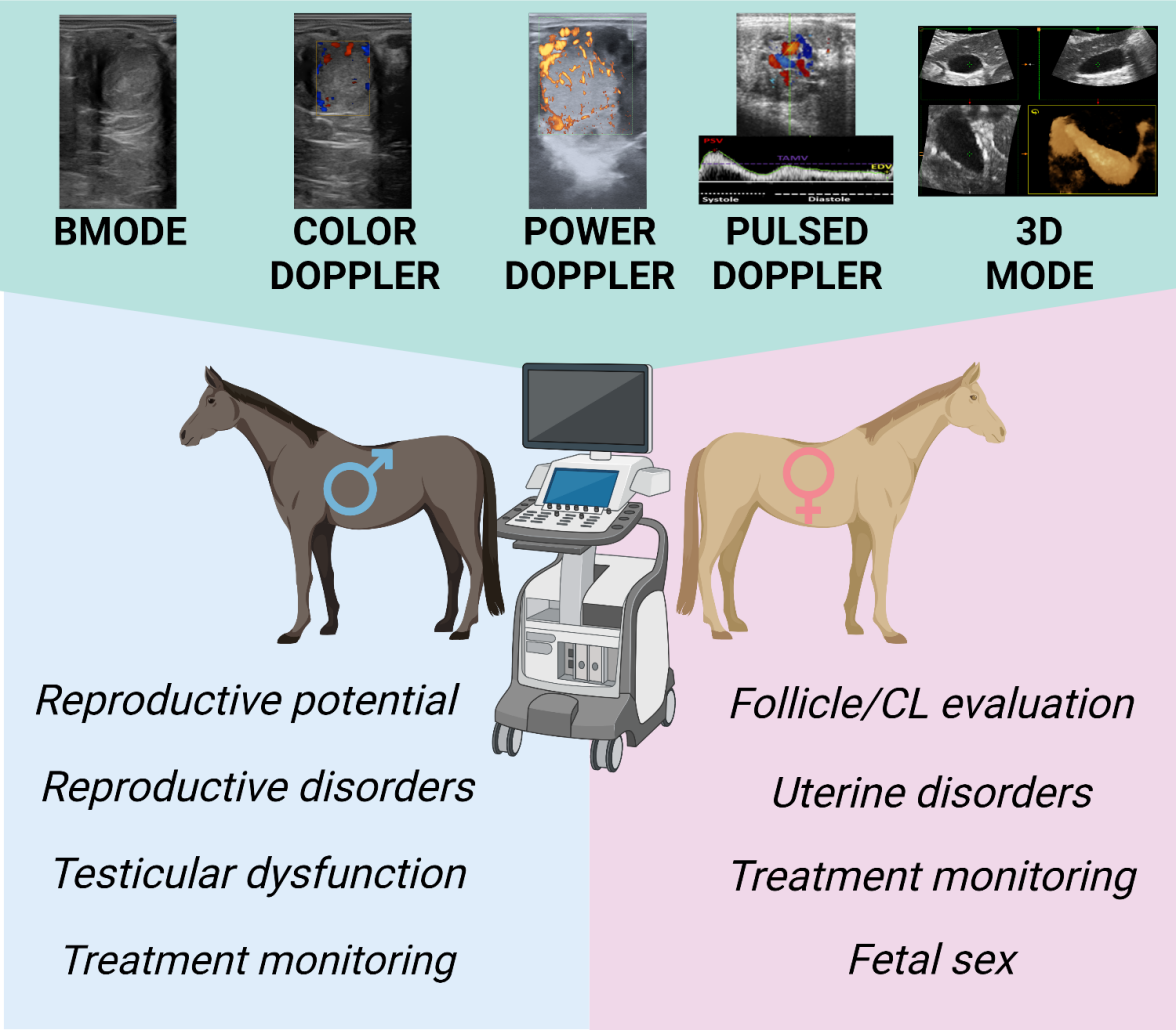
Summary figure. Advances in ultrasonography increased the clinical applications of this technique in equine reproduction

This review aims to discuss the potential clinical uses and new approaches of ultrasonography in equine reproduction (Figure 1, summary figure).

## 2 NEW APPLICATIONS OF DOPPLER ULTRASOUND IN MARES

Ultrasound has become the ‘gold standard’ tool in reproductive evaluation of the mare. Grey scale ultrasound has contributed to knowledge of physiological aspects of the mare such as characterization of follicular dynamics, the ovulation process and development of the (CL), or the phenomenon of embryo mobility (Acosta et al., 2004; Gastal et al., 2006a; Ginther, 2014; Ginther, Gastal, & Gastal, 2007). Moreover, Doppler ultrasound technology enables characterization of uterine and ovarian blood flow during the oestrous cycle and early pregnancy in mares (Bollwein et al., 2003; Bollwein, Weber, et al., 2002). In recent years, the incorporation of Doppler technology in routine clinics and its new applications has improved reproductive outcomes on farms.

### 2.1 Applications of Doppler ultrasound in artificial insemination programs

Blood flow assessment of both dominant follicles and CL has been widely used as an indirect measure of their functionality in several species including the mare (Acosta et al., 2004; Ginther, 2007; Ginther, 2014.).

#### 2.1.1 Follicular evaluation

The development of follicles is related to the formation of the vascular network in the theca interna. Previous studies showed that during follicular selection, dominant follicles had a greater area of blood flow in the wall than subordinate ones. Moreover, this greater vascularization occurs before changes in the follicular diameter were detected (Acosta et al., 2004). Monitoring follicular dynamics to predict the ovulation time for coordinating breeding or artificial insemination is one of the main clinical applications of ultrasound in routine reproductive practice (Ginther, 2007). The clinical signs of impending ovulation identified with B‐mode ultrasound are a deformed preovulatory follicle, increased thickness and echogenicity of the granulosa, and a reduction of the prominence of the anechoic band peripheral to the granulosa band (Miro, 2012). Colour Doppler ultrasound has also been used to predict ovulation time. Reductions in the percentage of the circumference with colour Doppler signals from the preovulatory follicle´s wall are observed during the period from 4 to 1 h before ovulation (Gastal et al., 2006b). In addition, the zona granulosa becomes serrated in association with extensive vascularization of the theca at the base of the follicle, or opposite the area of ovulatory rupture (Ginther, Gastal, & Gastal, 2007). A previous study has also shown that follicular size is not associated with vascularity, with follicular blood flow greater in old mares compared to young mares (Altermatt et al., 2012).

The identification of multiple ovulations (i.e. to prevent the carriage of twins) or timely detection of anovulatory follicles (i.e. transitional period) are crucial in the reproductive diagnosis. Pathological ovulation failure occurs in approximately 8.2% of oestrous cycles during the breeding season (Miro, 2012). Follicular diameter and echotexture assessed by grey scale ultrasound do not predict whether a follicle will ovulate or trigger a haemorrhagic follicle. The development of anovulatory haemorrhagic follicles is more common in the transition period in mares. Colour Doppler ultrasound enables the prediction of the development of anovulatory follicles because they present an increased vascularization, together with the apical area, which can be observed the day before ovulation failure with Doppler colour (Ginther, Gastal, Gastal, & Beg, 2007).

#### 2.1.2 CL evaluation

**FIGURE 2:**
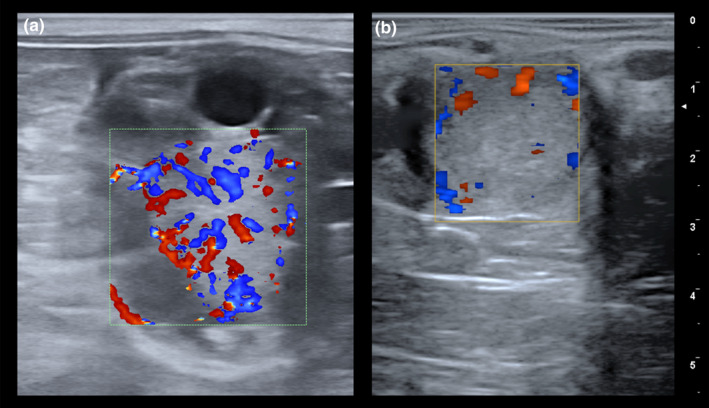
Colour Doppler evaluation of the corpus luteum (CL) in the mare. (a) Colour Doppler ultrasound image of a CL displaying a well‐established homogeneous pattern of vascularization, consistent with an active status of this gland. (b) Colour Doppler ultrasound image of a CL showing an absence of irrigation, indicative of a low functionality

Unlike the cow, the mare's CL is located within the ovary and does not protrude from the surface, so ultrasound is needed to identify it. It is estimated that 50% of CLs do not present a uniform echogenic image and may present a central anechoic cavity (Miro, 2012). No differences have been found in plasma levels of progesterone in heifers with cavitary CL (Jaśkowski et al., 2022). The CL is an endocrine gland characterized by a high vascular network. Colour Doppler ultrasonography is a fast, suitable method to evaluate CL functionality (Figure 2). Research has shown that there is a positive correlation between CL vascularization assessed with colour and power Doppler (area of coloured pixels) and levels of progesterone [P_4_] circulating in cycling mares. In addition, the vascularization of CL has proved to be a more dependable predictor of CL function than CL size, especially once luteal regression occurs (Bollwein, Mayer, et al., 2002; Ginther, Gastal, Gastal, Utt, & Beg, 2007). Both plasma progesterone levels and CL vascularization return to basal values 24 h after administration of prostaglandins. However, the size of the CL significantly decreases over the following 3 days (Miro, 2012). The CL vascularization and progesterone production have been also evaluated in Autumn and winter cycles in mares. The parameters of corpora lutea cross‐sectional area, vascularized area and index of vascularization (IV: VA/CSA) were significantly lower in the last cycles before anoestrus than in the first cycles of autumn, however, the level of progesterone among cycles did not change (Panzani et al., 2017).

### 2.2 Applications of Doppler ultrasound in embryo transfer programs

**FIGURE 3:**
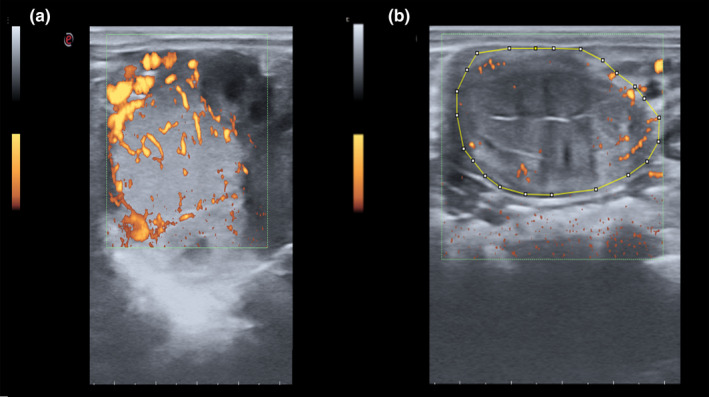
(a) Power Doppler ultrasound of a functional CL of 8 days postovulation in a mare. The day 8 postovulation is the day with the highest blood supply to the CL and with the highest production of progesterone. Power Doppler ultrasound is the most sensible technique to assess the vascularization of CL. (b) Endometrial blood flow evaluated with power Doppler ultrasound. Computer‐assisted pixel analysis using Image J software has been performed in research work in order to semi‐quantitatively evaluate the vascularization and echotexture of different organs based on the area and intensity of pixels identified in the image

In recent years, several studies have investigated the possibility of using Doppler imaging in embryo transfer programs, particularly in the selection of recipient mares and cows (Brogan et al., 2016; Pugliesi et al., 2018). The high sensitivity of Doppler ultrasound in the evaluation of CL functionality has promoted its use for the identification of recipients with good quality CL on the day of embryo transfer and thus with greater receptivity potential (Brogan et al., 2016; Ferreira et al., 2020) (Figure 3a). A recent study also showed that spectral Doppler measurement of the blood flow of the uterine arteries is an indirect way to the evaluation of CL functionality and thus a helpful tool for the selection of recipient mares for ET. Mares with RI values near 1.0 are correlated with mares with high CL vascularization and elevated P4 plasma concentrations (Ferreira et al., 2020). The relation between the area of colour flow Doppler of CL and progesterone concentrations has been also evaluated in mares after embryo transfer (Brogan et al., 2016). Results of this research suggest that both CL area and blood flow are correlated with circulating [P4] at the time of transfer and in early pregnancy.

In a previous study from our lab, we evaluated the clinical application of power Doppler ultrasound in the diagnosis of early pregnancy before embryo collection (Nieto‐Olmedo et al., 2020). To determine whether differences in uterine blood flow between pregnant and non‐pregnant mares can be used to predict the presence of the equine embryo before flushing in an embryo transfer program, power Doppler ultrasonography was used on a total of 52 mares on days 7 or 8 post‐ovulation. Previous studies have shown that embryo migration may cause localized effects on endometrial vascularization, causing stimulation of uterine blood flow (Bollwein et al., 2003). Power Doppler ultrasound and post‐acquisition image processing with Image J v1.48 software (computer analysis of Doppler images) were used, and significant differences were detected in the uterine blood flow area between pregnant and non‐pregnant mares (Figure 3b) (Nieto‐Olmedo et al., 2020). An increase in uterine blood flow area was observed in mares with positive flushings (one embryo: 54.01 ± 2.27 mm^2^ or two embryos: 61.01 ± 6.73 mm^2^) in comparison to barren mares (21.77 ± 2.22 mm^2^) (*p < .05*). This research showed blood flow to be a good predictive value to differentiate between pregnant and non‐pregnant mares with an AUC: 0.869; *p < .001* and an optimal cut‐off value of 37.21 mm^2^. These results suggest that Doppler is an accurate predictive method for early pregnancy diagnosis in mares (Nieto‐Olmedo et al., 2020).

### 2.3 Applications of Doppler ultrasound in the diagnosis of uterine disorders in mares

Endometritis is one of the most common causes of subfertility in mares. B‐mode ultrasound allows the identification of clinical signs of endometritis such as uterine fluid accumulation, endometrial hyperoedema, or categorization of the grade of the echogenicity of uterine fluid. Recently, colour and pulse Doppler ultrasound has been used in the diagnosis of endometritis. Mares with endometritis showed greater uterine vascularization (increased Doppler velocities and a significant decrease in Doppler indices) when compared to healthy mares (Abdelnaby et al., 2020). In addition, spectral Doppler was a suitable tool in the diagnosis of mares susceptible to persistent breeding‐induced endometritis (PBIE) before they were inseminated. Susceptible mares had blood flow velocity (BFV) values that were higher than in resistant mares even 1 day before the insemination, and a higher PI at 2 days after insemination (Lüttgenau et al., 2021).

In a preliminary study of our lab, we have evaluated for the first time the use of power Doppler ultrasound (PD) in combination with computerized image analysis as a marker of endometritis in mares and jennies (da Silva‐Álvarez et al., 2022). Both species showed an increase in endometrial BFA in pathological uterine conditions compared to controls. This parameter showed a good prognostic value, presenting an AUC of 0.94 (oestrus) and 0.98 (dioestrus), and using Youden's index, the cut‐off value for this parameter was also determined in mares (oestrus: 1.685 and dioestrus: 0.983). In jennies, the measurements were unified obtaining an AUC of 0.91 and a single cut‐off value of 2.970) (*p < .0001*). Several studies have suggested that age increases the incidence of uterine angiopathies in mares and could have important implications for endometrial glandular development, post‐breeding endometritis and early pregnancy death in older mares (Nambo et al., 1995; Oikawa et al., 1993). Transrectal colour and power Doppler ultrasonography have been used to study uterine blood flow and perfusion in mares with and without uterine cysts. Reduced uterine vascular perfusion was detected in mares with uterine cysts. Moreover, a positive association between the size of the cystic area and disturbances in uterine hemodynamics was established (Ferreira et al., 2008).

### 2.4 Applications of Doppler ultrasound in the monitoring of treatment in mares

Monitoring of medical treatment has also been performed in mares using Doppler ultrasound. There have been various studies in mares where treatments aimed at improving uterine and ovarian perfusion have been evaluated, including L‐arginine supplementation and pentoxifylline or the local administration of autologous platelet‐rich plasma (PRP) to treat acute endometritis (Bailey et al., 2012; Farghali et al., 2022; Kelley et al., 2014; Mesa et al., 2015).

### 2.5 Application of ultrasound for the diagnosis of fetal sex

**FIGURE 4:**
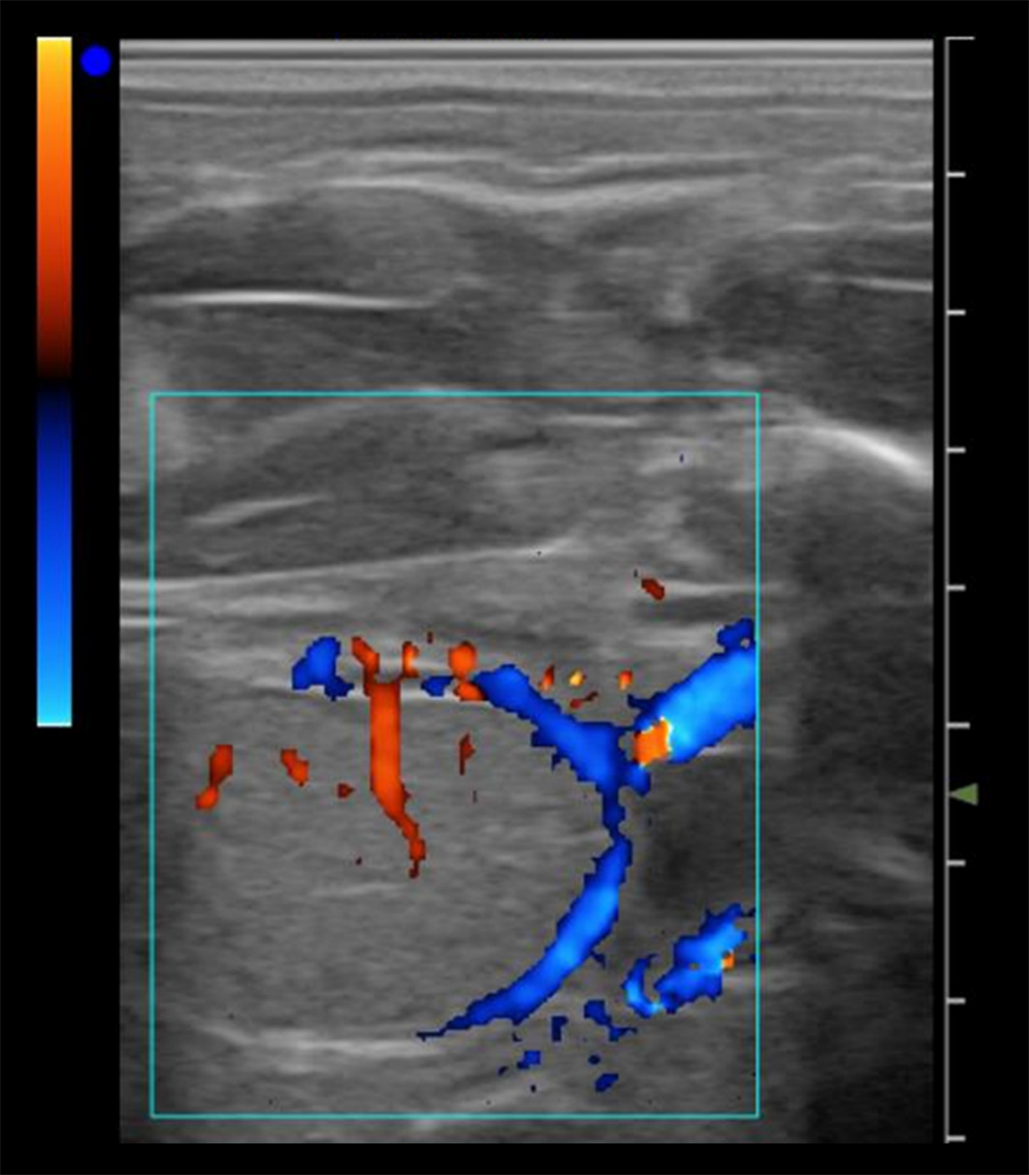
Determination of fetal sex by Doppler ultrasound. Colour Doppler ultrasound image at day 240 of gestation, showing the vascularization pattern characteristic of the male gonad, with the pampiniform plexus and the testicular vein

The diagnosis of equine fetal sex is a clinical activity that is increasingly in demand in veterinary practice. Sexing of the fetus can be performed during three specific timeframes: early (57–70 days), mid‐gestation (90–150 days), and late‐gestation (150–210 days). In the early sexing period (57–70 days), the determination of the sex is based on the location of the genital tubercle (precursor of either the male penis or the female clitoris) using transrectal B‐mode ultrasound (Curran & Ginther, 1989). However, a large amount of allantois fluid, fetal movements and the extremely long umbilical cord can make it difficult to identify and this procedure requires the practitioner to be experienced. Determination of equine fetal sex during mid‐gestation (90–150 days) using a B‐mode scan is based on the identification of fetal gonads and external genitalia (Bucca, 2005). Recent reports have demonstrated that colour Doppler ultrasonography combined with B‐mode ultrasonography provided greater accuracy in the diagnosis of fetal sex in both periods (mid and late‐gestation), allowing better visualization of the fetal gonads, especially in males (Mebarki et al., 2019; Resende et al., 2014) (Figure 4). The assessment of vascularization of the gonads using colour Doppler is a helpful tool for the non‐experienced practitioner for sexing of the fetus between 120 and 150 days. The pampiniform plexus or testicular vein in the internal gonads of the male fetus or visualization of the vascular ring between the cortex and the medulla of the ovaries are easily detected between 90 and 180 days of pregnancy (Resende et al., 2014).

Three‐dimensional (3D) ultrasound (US) imaging for assessing the equine fetus has also been used in mares in recent years. This technique was shown to be more accurate in the diagnosis of fetal sex determination than 2D‐ultrasound. Real‐time 3DUS could be successfully applied for transrectal examination of pregnant mares obtaining higher quality details of the genital tubercle (63‐ to 76‐day interval) and of external genitalia (90‐ to 150‐day interval) (Kotoyori et al., 2012; van de Velde et al., 2018). In addition, 3D US allows the determination of fetal volume in early pregnancy, providing an alternative to conventional measurements of rump length, eye diameter or aorta to estimate fetal growth (Becsek et al., 2019).

## 3 NEW CLINICAL APPLICATIONS OF ULTRASOUND IN STALLIONS

Ultrasound scanning is a non‐invasive diagnostic tool, established as the main diagnostic imaging technique for evaluation of the reproductive potential of the stallion, as well as in the diagnosis of testicular disorders and monitoring of medical and surgical treatments.

### 3.1 Evaluation of potential reproductive capacity

Breeding Soundness Evaluation (BSE) of a stallion is a service in high demand at the beginning of the reproductive season and in pre‐purchase examinations. B‐mode ultrasound makes predictions of sperm production capacity of a stallion from an estimation of testicular volume (TV) (TV: 0.053 × W × H × L) and calculation of the estimated Daily Sperm Output (eDSO: 0.024 × TTV‐0.76) (Love et al., 1991). Normally, when the actual sperm production (aDSO: volume of ejaculate × concentration) is well below the predicted one, this confirms that the stallion suffers from testicular dysfunction (Ortega‐Ferrusola et al., 2014). Moreover, the spermatogenic efficiency (number of sperm produced per unit of testis, aDSO/ml of the testis) is the criteria most widely used by practitioners for the diagnosis of testicular degeneration (Blanchard et al., 2001). Three‐dimensional sonography has been also performed recently in stallions to measure TV and predict DSO, being a more accurate method in determining TV than calliper measurement and B‐mode ultrasound (Pricking et al., 2017).

The evaluation of testicular echotexture by B‐mode ultrasound has been also used to predict semen quality in other species such as ram or bull (Ahmadi et al., 2013; Brito et al., 2012; Giffin et al., 2009).

### 3.2 Diagnosis of various pathological disorders in stallions

**FIGURE 5:**
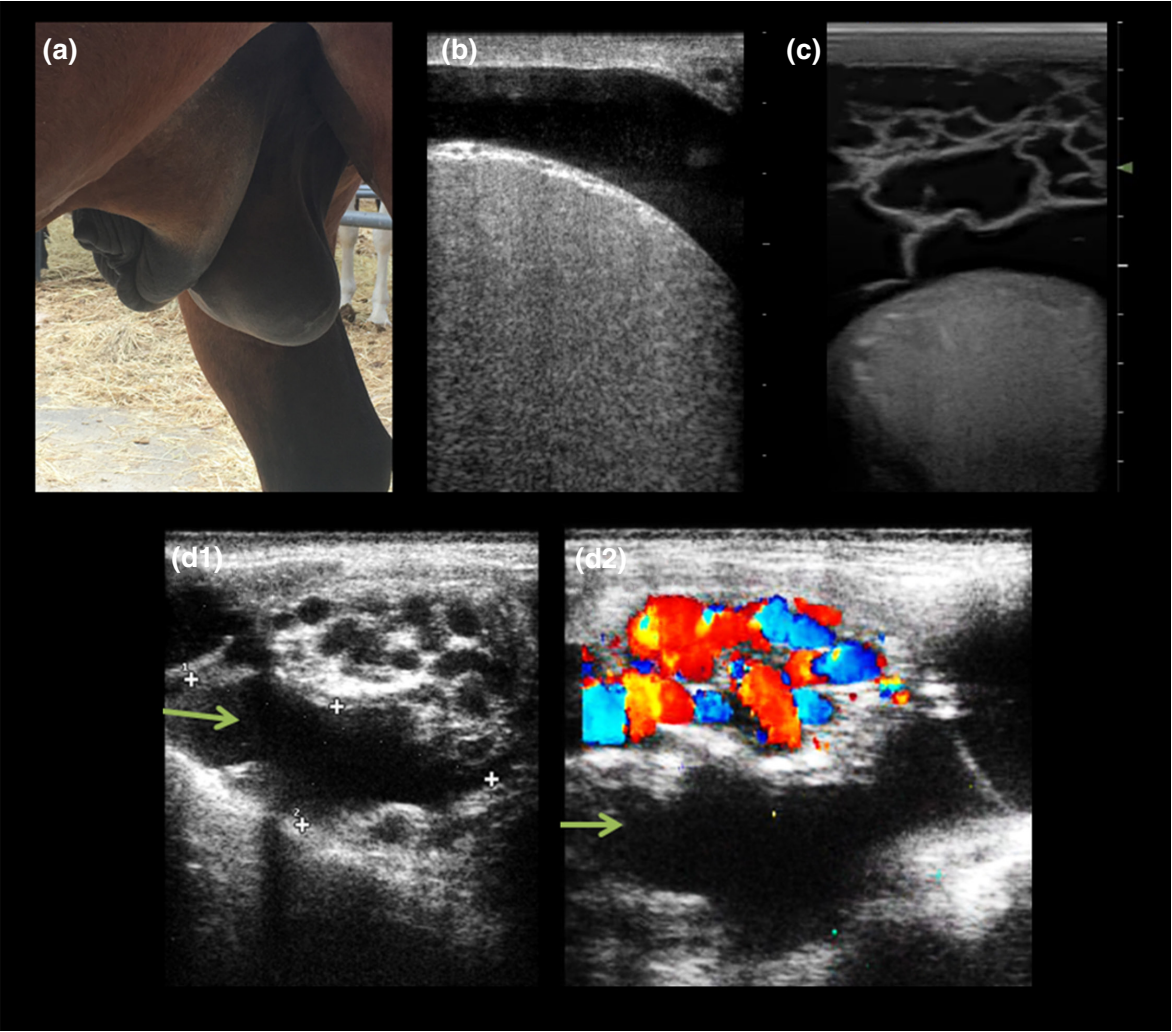
Pathological findings in male reproductive ultrasound. (a) External genitalia from a stallion with heart failure causing ventral oedema. Such tissue swelling could cause problems with the thermoregulation of the testicles as well as mechanical problems when externalizing the penis. (b) B‐mode ultrasound image showing clear anechoic fluid around the testicle, a condition known as hydrocele. (c) B‐mode ultrasound image displaying a cavity with trabeculae between the scrotum and the testicle. This kind image is characteristic of the haematocele. (d) Combination of b‐mode (D1) and colour Doppler ultrasound images of the testicular cord showing a tortuous dilated vein full of fluid (green arrows) but without signs of pulsatility

Previous studies from our laboratory have demonstrated the importance of evaluating testicular vascular perfusion as part of the diagnostic workup during the BSE, as well as in stallions with a history of subfertility, reproductive pathologies or abnormal spermiograms (Ortega‐Ferrusola et al., 2014; Ortiz‐Rodriguez et al., 2017). Ultrasound is a helpful tool for the diagnosis of several pathological disorders because it allows detection of not only changes in testicular volume but also provides information about anatomical structures, echotexture, and location of lesions (Morresey, 2007). B‐mode ultrasound is used for the diagnosis of extratesticular pathologies such as varicocele, haematocele, hydrocele or inguinal hernia (Henry et al., 2000; Love, 1992; Pozor, 2005; Schambourg et al., 2010) (Figure 5). Varicose veins are mainly seen peripherally around the spermatic cord and appear as dilated irregular anechoic structures with no signs of Pulsatility (Figure 5). To date veins with varicocele have been reported with a range of diameters between 8 and 24 mm (Pozor, 2005). Diagnosis of intratesticular lesions such as neoplasia or testicular degeneration used to be less specific and most of the time required additional techniques such as testicular biopsy (Brinsko, 1998; Edwards, 2008). Finally, conventional ultrasound is also used as a complementary technique to diagnose cryptorchid testicles (Jann & Rains, 1990). The identification of one or both affected testicles can be performed abdominally or rectally (Gracia‐Calvo et al., 2014).

Colour and spectral Doppler ultrasound has also been proposed as a helpful tool to discern between some pathological conditions such as epididymal‐orchitis and testicular torsion (Pozor, 2005; Pozor & McDonnell, 2004). In stallions, epididymorchitis triggers a decrease in RI due to hyperaemic processes. In contrast, in ischemic processes, the RI values increase due to major vascular resistance (Pozor & McDonnell, 2004). Interruption of blood flow within the testicular parenchyma characterizes acute cord torsions. Clinical signs and how this is visualized in images produced by Doppler ultrasound are dependent on the level of twisting and for how long torsion has been present. Clinical signs may not be present in torsions of <180°. With torsions up to 180°, venous flow is cut‐off and venous dilation may be seen but the arterial flow is still present. In cases of 180° spermatic cord torsion in horses early diastolic retrograde blood flow in the supratesticular artery has been seen (Pozor & McDonnell, 2004).

High values of Doppler indices have also been described in cases of severe hydrocele and chronic non‐strangulated inguinal hernia (Gracia‐Calvo et al., 2015; Samir et al., 2021). Any reduction in the blood flow to the testis (herniation, testicular torsion, or varicocele) causes ischemic damage in the seminiferous tubules and can affect sperm production and quality.

### 3.3 Diagnosis of testicular dysfunction

Stallions of all ages can be affected by testicular dysfunction, which can cause temporary subfertility, and this can lead to irreversible testicular degeneration depending on the initial cause and degree of damage. Thus, early detection of signs of testicular dysfunction is crucial to implement timely treatment and recover the fertility of these stallions.

As mentioned before, grey scale ultrasound allows the calculation of testicular volume and spermatogenic efficiency (DSO per millilitre of testis) in a stallion. However, testicular volume is often only affected by the majority of pathologies in the later stages of the disease and no change in testicular echogenicity is visually detectable (Turner, 2005).

In several different species, computer‐assisted pixel analysis has been applied to quantify the echotexture of testicular parenchyma, providing an alternative method for the evaluation of testicular tissue architecture. Variations in these parameters have been correlated with changes in testicular tissue histomorphology and semen quality (Arteaga et al., 2005; Brito et al., 2012; Ahmadi et al., 2013; Giffin et al., 2014).

**FIGURE 6:**
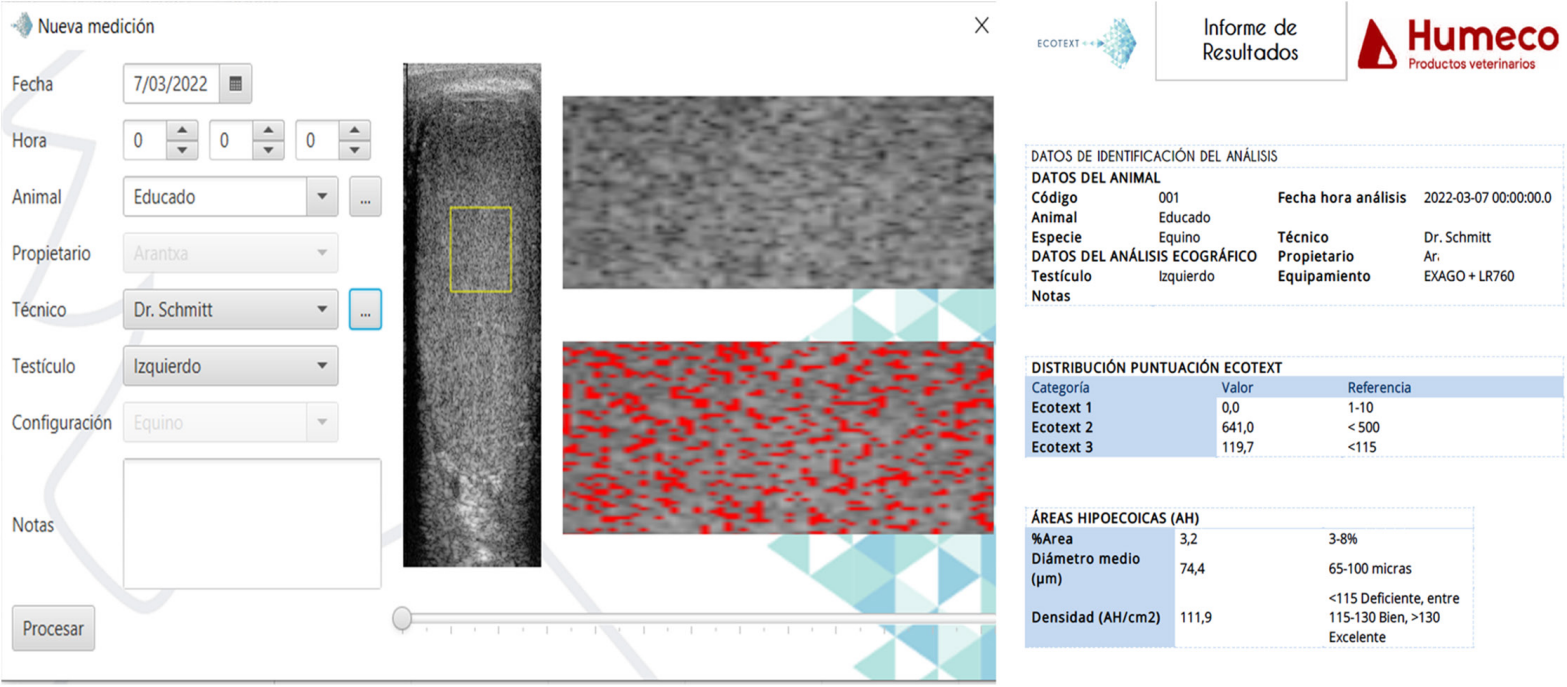
Computer testicular echotexture analysis using Ecotext software in stallions. This software (Ecotext, Humeco, Spain) is a diagnostic method of testicular dysfunction in stallions. According to the manufacturer, Ecotext provides the user with a total of six different parameters: Three parameters at normal resolution: Ecotext 1, Ecotext 2 and Ecotext 3, related to the number of black, white, and grey pixels respectively, and three parameters at high resolution related to hypoechogenic areas: Ecotext tubular density (density of hypoechogenic areas), Ecotext tubular diameter (mean diameter of hypoechogenic areas) and Ecotext tubular area (total percentage of hypoechogenic area)

Recently, our group has evaluated a new specially developed software (Ecotext, Humeco, Spain) for testicular echotexture analysis as a diagnostic method of testicular dysfunction in stallions (da Silva‐Álvarez et al., 2022). Ecotext provides the user with a total of six different parameters (Ecotext 1 (black pixels), Ecotext 2 (white pixels) and Ecotext 3 (grey pixels), Ecotext tubular density (density of hypoechogenic areas), Ecotext tubular diameter (mean diameter of hypoechogenic areas) and Ecotext tubular area (total percentage of hypoechogenic area), that were used as functionality markers (Figure 6). Ecotext can be used to identify changes in the testicular echotexture of stallions with testicular dysfunction, with lower values for Ecotext 1, Ecotext tubular area and Ecotext tubular density found in these stallions than in control stallions. Moreover, we found differences in Echotexture among stallions with acquired chronic TD and immunized stallions (da Silva‐Álvarez et al., 2022).

Doppler ultrasound is a good tool for the early diagnosis of testicular dysfunction. In a preliminary study by our research group in which testicular dysfunction was induced through anti‐GnRH immunization, we were able to verify that immunocastration with Improvac® reduced blood flow to the testis before sperm quality and production were affected. Testicular vascularization (TABF) significantly decreased 1 month after the first Improvac® vaccine was administered. However, all quality and production parameters evaluated significantly decreased 2 months after the first vaccine. Once again, this highlights the importance of adequate irrigation for the correct functioning of the testicle.

Adequate testicular vascularization is essential to support the metabolic requirements of the testes necessary to carry out spermatogenesis and the synthesis of hormones. Several studies have demonstrated that Doppler indices are potential markers of testicular functionality since Doppler parameters are correlated with sperm quality and production parameters (Biagiotti et al., 2002; Gloria et al., 2018; Pozor et al., 2014; Zelli et al., 2013). In reproductive medicine, RI is a marker of testicular dysfunction widely used for the identification of patients suffering from different causes of testicular dysfunction (Pinggera et al., 2008). In oligospermic stallions and those with pharmacologically induced testicular dysfunction, the RI was also found to be increased (Ortiz‐Rodriguez et al., 2017; Pozor et al., 2014), which indicates that this may be a potential method which could be used to diagnose and differentiate obstructive azoospermia (blockage of the ampulla) from the non‐obstructive type. Previous reports from our lab revealed that the best Doppler parameters to predict sperm quality in stallions were: Doppler velocities (PSV, EDV and TAMV), the diameter of the capsular artery and TABF parameters (tissue perfusion parameters) (Ortiz‐Rodriguez et al., 2017). Stallions with poor semen quality with low sperm production and motility are characterized by an increase in Doppler indices and a decrease in Doppler velocities, with testicular artery (capsular artery) diameters below 2.9 mm (Ortiz‐Rodriguez et al., 2017). Cut‐off values were established for the first time to differentiate between fertile stallions and those with chronic testicular dysfunction. In this study, important correlations were found between Doppler parameters and the quality of refrigerated semen (sperm motility, viability, mitochondria and DNA fragmentation) (Ortiz‐Rodriguez et al., 2017). This makes sense, since ischemic processes at the testicular level would produce an increase in reactive oxygen species (ROS), triggering sublethal damage to spermatozoa, making them more susceptible to apoptosis and less tolerant to refrigeration and freezing (Bergh et al., 2001).

### 3.4 Monitoring of therapeutic outcomes after medical or surgical treatment in stallions

In recent years, different treatments have been investigated to improve testicular blood flow after vascular disturbance and preserve the functionality of the testes. In stallions, the administration of a single dose of hCG (5000 IU) or the oral administration of pentoxifylline increased testicular blood flow in treated stallions (Bollwein et al., 2008; Pozor et al., 2011). Pentoxifylline treatment could be a useful medical therapy for several testicular conditions involving impaired microvascular perfusion (Oliva et al., 2009).

Doppler ultrasound has also allowed monitoring of surgical techniques. Our research group carried out a study evaluating whether the preventive closure of the inguinal rings using standing laparoscopic peritoneal flap hernioplasty (SLPFH) compromised testicular vascularization and the seminal quality of these horses. In this study, pulsed Doppler ultrasound and conventional seminal evaluation techniques were performed in order to check whether reproductive function of stallions was preserved after this intervention (Gracia‐Calvo et al., 2014; Gracia‐Calvo et al., 2015). Sperm production and sperm quality parameters of stallions on which surgery was performed were not affected during the year after surgery. However, curiously, it was observed that all the stallions presented a decrease in Doppler indices and an increase in Doppler velocities at 12 months after surgery (Gracia‐Calvo et al., 2015). Thus, the use of Doppler is a good tool for monitoring the therapeutic outcome after surgery and enables early detection of vascular alterations.

## 4 CONCLUSION

Doppler ultrasound evaluation should be included in the diagnostic work‐up of both mares and stallions. In stallions, it should form part of the BSO and be used as a diagnostic tool in all cases of scrotal disorders in clinical practice. The use of this tool allows early identification of testicular pathologies and improves treatment outcomes as a result of timely treatment administration. In mares, it provides a method for fast, reliable evaluation of CL and improved selection of recipient mares for embryo transfer, early pregnancy diagnosis as well as diagnosis of mares affected by endometritis or determining of fetal sex.

## AUTHOR CONTRIBUTIONS

All authors equally contributed in writing this review article.

## CONFLICT OF INTEREST

None of the authors have any conflict of interest to declare.

## Data Availability

Data sharing not applicable to this article as no datasets were generated or analysed during the current study.
